# Integrated Macrogenomics and Metabolomics Analysis of the Effect of Sea Cucumber Ovum Hydrolysates on Dextran Sodium Sulfate-Induced Colitis

**DOI:** 10.3390/md23020073

**Published:** 2025-02-07

**Authors:** Shunmin Gong, Liqin Sun, Yongjun Sun, Wenming Ju, Gongming Wang, Jian Zhang, Xuejun Fu, Chang Lu, Yu Zhang, Wenkui Song, Mingbo Li, Leilei Sun

**Affiliations:** 1 Yantai Key Laboratory of Characteristic Agricultural Bioresource Conservation & Germplasm Innovative Utilization, School of Life Sciences, Yantai University, Yantai 264005, China; g18807041394@163.com (S.G.); sliqin2005@163.com (L.S.); fuxuejun@126.com (X.F.); luchang0808@sina.com (C.L.); zhangyu1994@pku.edu.cn (Y.Z.); 2 Homey Group Co., Ltd., Rongcheng 264300, China; sun29889@163.com (Y.S.); jwm001506@163.com (W.J.); 3 Yantai Key Laboratory of Quality and Safety Control and Deep Processing of Marine Food, Shandong Marine Resource and Environment Research Institute, Yantai 264006, China; wgmsd105@163.com (G.W.); zjsd408@163.com (J.Z.); 4 Guangdong Provincial Key Laboratory of Aquatic Products Processing and Safety, National Research and Development Branch Center for Shellfish Processing (Zhanjiang), Guangdong Provincial Engineering Technology Research Center of Seafood, Guangdong Province Engineering Laboratory for Marine Biological Products, College of Food Science and Technology, Guangdong Ocean University, Zhanjiang 524088, China

**Keywords:** sea cucumber ovum hydrolysates, colitis, intestinal flora, microbial metabolism

## Abstract

Inflammatory bowel disease remains a significant challenge in clinical settings. This study investigated the therapeutic potential of sea cucumber ovum hydrolysates (SCH) in a dextran sulfate sodium (DSS)-induced colitis mouse model. SCH, defined by its elevated stability and solubility, with a molecular weight below 1000 Da, significantly alleviated DSS-induced colitis, as evidenced by enhanced splenic index, reduced colonic damage, and diminished serum pro-inflammatory cytokines. Furthermore, macrogenomic analysis demonstrated that SCH increased beneficial gut microbes and decreased pro-inflammatory bacteria. Furthermore, metabolomic analysis of colonic tissues identified elevated levels of anti-inflammatory metabolites, such as Phenyllactate, 2-Hydroxyglutarate, and L-Aspartic acid, in colitis mice after oral administration of SCH. In conclusion, SCH represents a promising candidate for the treatment of colitis.

## 1. Introduction

Ulcerative colitis (UC), a form of inflammatory bowel disease (IBD), is typically characterized by the destruction of the mucosal surface, bloody diarrhea, and a heightened susceptibility to the development of colorectal cancer (CRC) if left untreated [1,2,3]. It is generally accepted that UC is a multifactorial disease resulting from a combination of genetic, environmental, and immunological factors, as well as dysbiosis of gut microbiota. However, the precise etiology remains unknown. The current pharmacological treatments for UC, including 5-aminosalicylic acid and corticosteroids, are known to have significant side effects [4,5]. Therefore, there is an urgent need to develop highly effective, low-toxicity natural drugs or dietary interventions for the prevention and treatment of UC.

Recent evidence suggests that dysbiosis of intestinal flora plays a significant role in colonic inflammatory diseases [6]. It has been reported that the transfer of fecal microbiota from colitic mice to healthy mice induces colitis [7]. The levels of beneficial bacteria, such as *Bifidobacterium longum*, *Fusobacterium rectum*, *Enterobacter pulaefaciens*, and *Rose bacillus intestinalis*, were significantly reduced in the intestinal tracts of patients with UC compared to healthy individuals, whereas the relative abundance and growth rate of harmful bacteria, including *Anaplasma fragilis*, were increased [8]. The balance between beneficial and harmful bacteria is directly related to the integrity of the intestinal epithelial barrier and the function of immune cells. Therefore, targeting gut flora as a therapeutic strategy for UC is a promising approach.

Sea cucumbers have been utilized as both food and medicine for centuries, playing a significant role in agriculture, nutraceuticals, pharmaceuticals, and other industries [9]. Peptides derived from sea cucumbers, which can be prepared through microbial fermentation, chemical hydrolysis, and enzymatic hydrolysis, may exhibit potential anti-inflammatory properties due to their composition, which encompasses various non-essential and essential amino acids [10]. Recent studies have demonstrated that sea cucumber peptides can alleviate UC by regulating intestinal flora, and some insights have been gained regarding the microbiological mechanisms underlying the action of these peptides in the treatment of UC [11]. However, there remains a lack of comprehensive understanding concerning the mechanisms of action of microbial-derived metabolites. These metabolites are crucial for microorganisms to perform various biological functions within the gut. Disturbances in the gut metabolome have been observed in patients with IBD, characterized by imbalances in short-chain fatty acids (SCFA), bile acids, and tryptophan [12,13]. Therefore, based on the understanding of how sea cucumber peptides regulate intestinal flora, it is imperative to explore changes in the metabolome to identify potential targets related to microorganisms and their metabolites.

In our study, we utilized ovum from sea cucumber by-products to produce sea cucumber ovum hydrolysates (SCH) through enzymatic hydrolysis. We characterized its structural characteristics and physicochemical properties, and conducted in vitro simulated gastrointestinal digestion (SGD) to preliminarily assess its stability. Subsequently, we employed a colon colitis mouse model to evaluate the potential of SCH to alleviate colitis. Additionally, we analyzed the protective effects and underlying mechanisms of microorganisms and their metabolites through a combined analysis of metagenomics and metabolomics.

## 2. Results

### 2.1. Structural Characteristics and Physicochemical Properties of SCH and SCH-SGD

#### 2.1.1. Molecular Weight Distribution

The molecular weight distribution of SCH and SCH-SGD is presented in Table 1. SCH primarily comprised molecules in the <500 Da (70.24%) and 500–1000 Da (27.97%) ranges. Notably, no significant molecular weight distribution was observed in the SCH-SGD, with values of <500 Da (68.64%) and 500–1000 Da (30.49%). This indicated that there were no substantial changes in the molecular composition of the SCH-SGD. These findings suggested that SCH was predominantly composed of oligopeptides below 1000 Da and exhibited high stability following SGD, highlighting that resistance to gastrointestinal protease digestion was crucial for the bioactivity of peptides [14].

#### 2.1.2. Surface Hydrophobicity and Solubility

**Figure 1:**
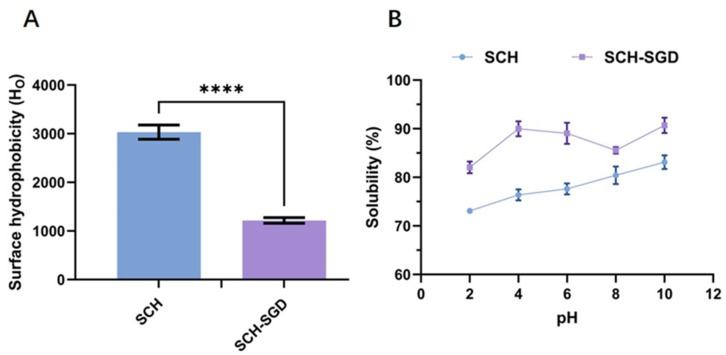
Surface hydrophobicity and solubility of SCH and SCH-SGD. (**A**) Surface hydrophobicity; (**B**) Solubility. **** *p* < 0.0001.

The degree of exposure of hydrophobic groups on the protein surface can be assessed through surface hydrophobicity, which reflects changes in protein structure [15]. The results indicated (Figure 1A) that the hydrophobicity of SCH-SGD was significantly reduced (*p* < 0.0001), probably due to pepsin’s preferential cleavage of proteins at the carboxyl side of hydrophobic residues, such as Phe and Leu, during gastric digestion [16]. Furthermore, solubility is a critical index for evaluating the biological activity of peptides, and the results demonstrated (Figure 1B) that the SCH-SGD exhibited superior solubility compared to SCH in both acidic and alkaline environments.

The combination of molecular weight results suggested that SCH, following in vitro SGD, might represent an active peptide characterized by a high degree of stability, dispersion, and solubility, thereby providing a theoretical basis for subsequent in vivo animal experiments.

### 2.2. SCH Alleviated the Symptoms of DSS-Induced Colitis

**Figure 2:**
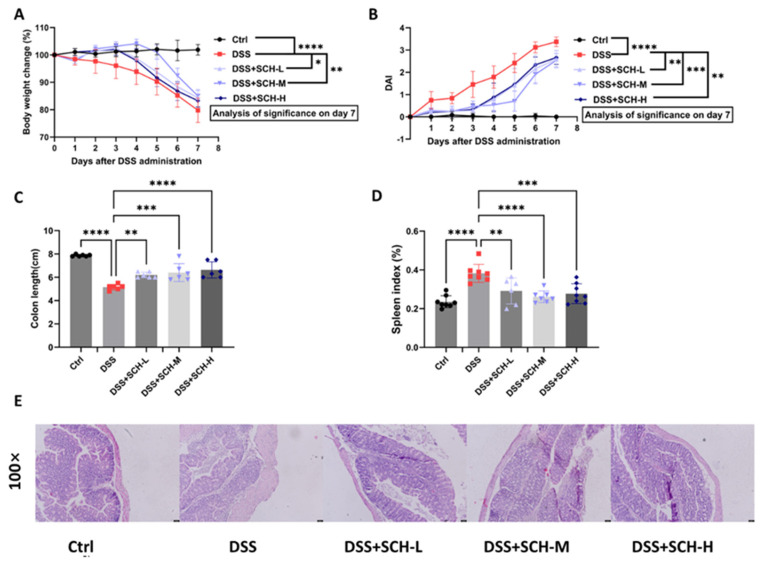
Effects of SCH administration on symptoms of DSS-induced colitis in mice. (**A**) Body weight change; (**B**) DAI scores; (**C**) Colon length; (**D**) Spleen index; (**E**) HE staining of the distal colon (magnification of 100×). * *p* < 0.05, ** *p* < 0.01, *** *p* < 0.001, **** *p* < 0.0001.

The objective of this study was to determine whether SCH exerted a prophylactic effect against colitis. To achieve this, a mouse model of colitis was subjected to treatment with 3% DSS, resulting in the manifestation of typical colitis symptoms, including weight loss, diarrhea, and the presence of blood in the stool. After 7 days of DSS administration, mice in the model group experienced a 21.2% loss in body weight compared to the control group. Notably, low and medium doses of SCH significantly mitigated this weight reduction in a dose-dependent manner (Figure 2A). In addition, DAI scores were significantly elevated following DSS treatment compared to the control group, reaching a peak of 3.38 ± 0.21 after 7 days (Figure 2B). In contrast, treatments with low-dose (SCH-L), medium-dose (SCH-M), and high-dose (SCH-H) SCH significantly prevented the elevated DAI scores to 2.58 ± 0.39, 2.54 ± 0.31, and 2.67 ± 0.31, respectively. Colonic shortening, a common symptom of colitis primarily resulting from colonic congestion, inflammation, and edema, was also observed. In this study, the colon length was significantly reduced in the DSS-treated group compared to the control group (7.87 ± 0.08 cm vs. 5.15 ± 0.23 cm, *p* < 0.0001). Treatment with SCH-L, SCH-M, and SCH-H significantly improved colon length to 6.20 ± 0.24, 6.40 ± 0.77, and 6.63 ± 0.70 cm, respectively (Figure 2C). Additionally, SCH treatment significantly alleviated DSS-induced splenomegaly and decreased the splenic index (Figure 2D). The assessment of colonic injury in mice utilizing histological HE staining revealed that control mice exhibited normal colonic morphology characterized by abundant cup cells, intact mucosa, and a thin muscle layer. However, the colons of DSS-treated mice displayed significant damage, as evidenced by marked damage to the epithelial surface, absence of colonic cells in the cups, deformation of the crypts, thickened edema in the muscularis propria, and enhanced infiltration of inflammatory cells (Figure 2E). Furthermore, the histological features, which included slight mucosal ulceration, damage to the crypts, edema, and infiltration of small cells into the mucosal tissue, were ameliorated by the administration of SCH. In conclusion, our data suggested that treatment with SCH significantly alleviated the symptoms of DSS-induced colitis in mice.

### 2.3. Anti-Inflammatory Effects of SCH

**Figure 3:**
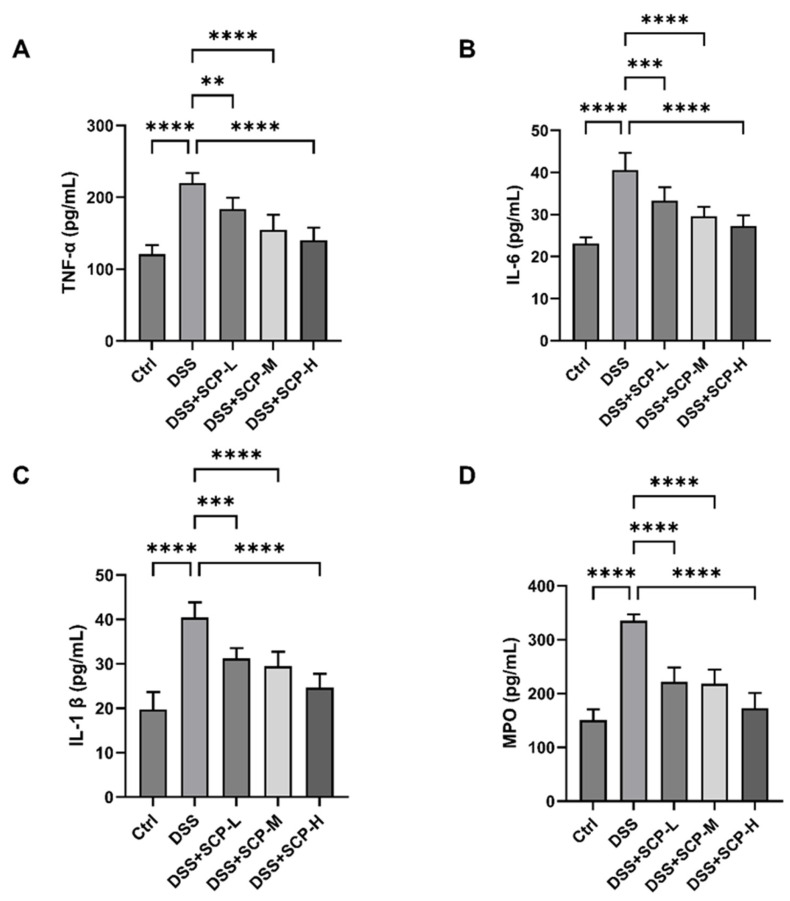
Effects of SCH administration on pro-inflammatory factors and MPO secretion in both serum and colonic tissues. (**A**) TNF-α; (**B**) IL-6; (**C**) IL-1β; (**D**) MPO. ** *p* < 0.01, *** *p* < 0.001, **** *p* < 0.0001.

The role of pro-inflammatory cytokines has been extensively studied, and their dysregulation is believed to play a significant role in the pathogenesis of IBD [17]. IL-1β, the prototypical pro-inflammatory cytokine belonging to the IL-1 family, is a central player in IBD pathogenesis. TNF-α and IL-6, which contribute to the intestinal inflammatory process, are also major cytokines involved in IBD pathogenesis [18,19,20]. In our study, serum levels of TNF-α, IL-6, and IL-1β were examined in mice. The levels of TNF-α, IL-6, and IL-1β were significantly increased in the DSS group compared to the control group (*p* < 0.0001), with increases of 56.57%, 50.55%, and 72.92%, respectively. However, a trend of dose-dependent decrease in all three pro-inflammatory factors was observed following SCH treatment (Figure 3A–C). In addition, we assessed the activity of MPO in the colon tissues, with results presented in Figure 3D. MPO activity, a key indicator of neutrophil infiltration [21], was significantly elevated in the DSS group. Natural zinc-enriched oyster peptide significantly reduced MPO activity in the colonic tissues of DSS-induced colitis mice [22]. Additionally, a dose-dependent reduction in MPO activity was observed in SCH-treated mice. Notably, MPO activity decreased by 49% in the SCH-H group compared to the DSS group, suggesting that SCH inhibited the excessive proliferation of MPO-positive cells and neutrophil infiltration in the tissues.

### 2.4. SCH Modulated the Gut Microbiota

**Figure 4:**
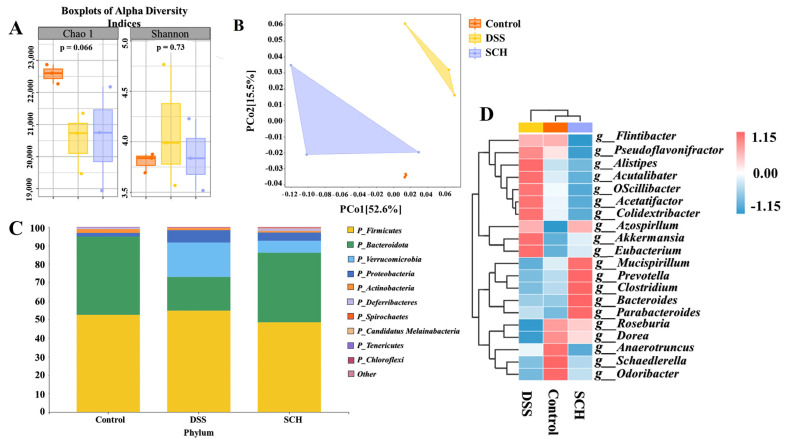
Effects of SCH administration on gut microbiota in mice with DSS-induced colitis. (**A**) α-diversity indices (Shannon and Chao 1 indices); (**B**) Principal coordinate analysis; (**C**) Species composition at the phylum level; (**D**) Heatmap of species composition at the genus level.

Fecal macrogenome sequencing was employed to monitor the effects of SCH use on the fecal gut microbiota of mice with colitis. The α-diversity indices, specifically the Shannon and Chao 1 indices, did not show significant differences among the groups (Figure 4A). These findings indicated that the administration of SCH did not result in substantial changes in the number or diversity of gut flora in colitis mice, suggesting that the observed improvement in colitis symptoms was not attributable to drastic alterations in the gut microbiota. Therefore, we further analyzed the changes in the gut microbiota at a more refined taxonomic level. Principal coordinate analysis, utilizing Bray–Curtis distance as illustrated in Figure 4B, was performed to assess the differences in fecal microbiota among the groups. The results revealed that the intestinal flora structure of DSS-induced colitis mice differed significantly from that of control mice not treated with DSS, as well as from the SCH group. SCH supplementation resulted in dramatic changes in the intestinal flora structure of colitis mice. The mice fecal microbiota was subsequently taxonomically annotated, revealing that the *Bacteroidetes* and *Firmicutes* phyla were dominant across all groups, accounting for over 70% of the total gut microbiota (Figure 4C). Compared to control mice, those in the DSS group exhibited a decrease in *Bacteroidetes* and an increase in *Firmicutes*, yet SCH supplementation reversed this unfavorable trend. To further elucidate the specific differences in microbial composition among the three groups, we annotated the top 20 microorganisms in terms of genus-level abundance (Figure 4D). The DSS group exhibited an increase in the amount of *Pseudoflavonifractor*, *Alistipes*, *Acutalibacter*, *Oscillibacter*, *Acetatifactor*, *Colidextribacter*, *Akkermansia*, and *Eubacterium*, while the amount of *Roseburia*, *Dorea*, *Anaerotruncus*, *Schaedlerella*, and *Odoribacter* was reduced compared to the control group.

To identify specific bacterial species within the three groups, we analyzed the macrogenomics data using Linear Discriminant Analysis Effect Size (LEfSe). The core genera identified in the control group included *p-Antinobacteria*, *g-Anaerotruncus*, *c-Coriobacteriia*, and *g-Muribaculum*. In contrast, the core composition of intestinal colonies in colitis mice comprised *s-Escherichia coli*, *g-Acutalibacter*, and *s-Duncaniella freteri*.

**Figure 5:**
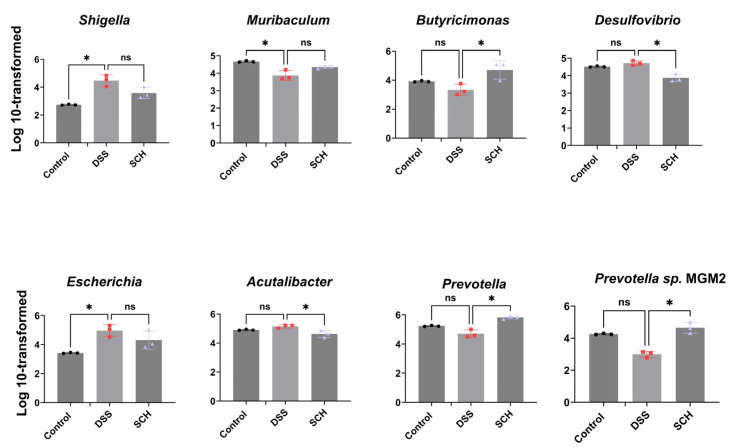
Effects of SCH administration on gut microbiota composition. Genus and species level intergroup differences species. * *p* < 0.05 and “ns” denotes no statistically significant difference.

Additionally, we conducted significance analyses for specific flora that exhibited differences between groups. We performed a Log 10 transformation of the abundance of species at the genus level and subsequently tested the transformed data for significance between groups using the Kruskal-Wallis test. The results were presented in Figure 5, which indicated that the DSS group exhibited a significant increase in the amount of *Shigella* and *Escherichia*, alongside a decrease in the amount of *Muribaculum* when compared to the control group. The role of SCH reversed this trend, albeit not to a significant extent. Furthermore, SCH significantly reduced the amount of *Desulfovibrio* and *Acutalibacter*, while markedly increasing the amount of *Butyricimonas* and *Prevotella*, along with its offshoot *Prevotella* sp. MGMZ. These changes may represent the anti-inflammatory mechanism associated with SCH.

### 2.5. SCH Modified the Gut Functional Microbial Metagenome

**Figure 6:**
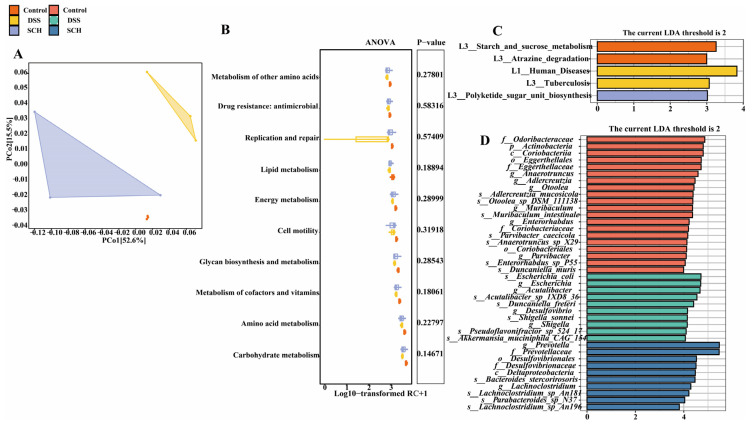
Effects of SCH administration on the gut functional microbial metagenome in mice with DSS-induced colitis. (**A**) β-diversity analysis at the KO level; (**B**) Multi-group analysis of differences in the KEGG pathway at level two; (**C**) LEfSe analysis of KEGG functions at level three; (**D**) LEfSe analysis of gut microbial composition across three groups.

Changes in the structure of the intestinal flora are invariably accompanied by alterations in the function of the intestinal microflora. Therefore, we further investigated the functional consequences of SCH supplementation. Bray–Curtis Principal Coordinates Analysis (PCoA) based on Kyoto Encyclopedia of Genes and Genomes (KEGG) immediate homologue levels (KO) demonstrated significant separation between groups (Figure 6A). At the level of 2-level functional differences in KEGG, our results indicated that among the ten pathways most enriched for genes, the function of normal mice gut microbes was diminished in the presence of DSS. However, this trend was reversed following SCH intervention across all pathways (Figure 6B). It is important to note that not all pathways exhibited significant differences. We further employed LEfSe analysis at the three levels of KEGG function to identify four metabolic pathways, consistent with LDA > 2, which revealed that the metabolic pathways in the control group included starch and sucrose metabolism as well as atrazine degradation (Figure 6C).

### 2.6. SCH Regulated the Metabolome of the DSS-Induced Colitis Mice

**Figure 7:**
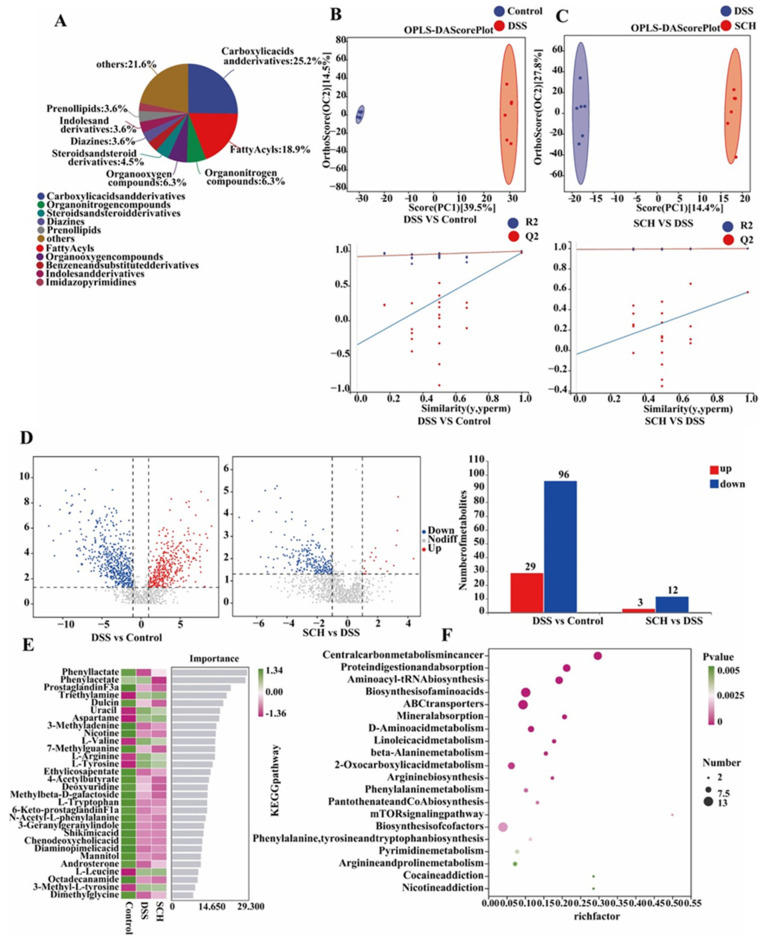
Effects of SCH administration on microbial metabolites in mice with DSS-induced colitis. (**A**) Percentage of each microbial metabolite; (**B**,**C**) Discriminant analysis of orthogonal projections of latent structures along with permutation tests; (**D**) Volcano plots highlighting differential variables and bar graphs illustrating the impact of SCH intervention on microbial metabolites in DSS-induced colitis mice; (**E**) Top 30 metabolites identified through machine learning analysis across the three group comparisons; (**F**) Pathway enrichment analysis of the three groups.

In this study, metabolomic analyses were performed on the colon tissue of each mouse. The results indicated that 142 metabolites were detected in positive ion mode and 69 metabolites in anion mode. The top five metabolites identified were carboxylic acids and derivatives, fatty acyls, organonitrogen compounds, organooxygen compounds, and steroids and steroid derivatives, which collectively accounted for 25.2%, 18.9%, 6.3%, 6.3%, and 4.5% of the overall metabolite count, respectively (Figure 7A). The metabolomics data of the DSS group were compared with those of the control and SCH groups to observe the alterations in metabolites in normal mice induced by DSS, as well as the effects of SCH on the metabolites of DSS-induced mice (Figure 7B,C). The metabolomic datasets for the control and SCH mice, along with the DSS group, exhibited a distinct clustering pattern, indicating significant differences in the metabolomics of colonic tissues between colitis mice and both normal and SCH-intervened colitis mice. Furthermore, the metabolomics of the mice were analyzed using a partial least squares discriminant analysis model to elucidate the impact of SCH on gut microbial metabolism. All Q2 points obtained from the model were lower than the original Q2 points on the far right, indicating that the model possessed good accuracy and predictability. In addition, DSS induced a significant up-regulation of 29 metabolites, including triethylamine, methylmalonic acid, and uracil, along with a significant down-regulation of 96 metabolites, such as diaminopimelic acid, 7-methylguanine, and octadecanamide. Subsequently, we employed differential substance mechanistic learning analyses to further identify marker differential metabolites among the top-ranked groups, as illustrated in Figure 7E. The metabolites phenyllactate, phenyl acetate, uracil, and 7-methylguanine emerged as the highest-ranked. These findings suggested that SCH enhanced the production of anti-inflammatory compounds. Furthermore, pathway enrichment analysis revealed that central carbon metabolism, protein digestion and absorption, aminoacyl-tRNA biosynthesis, biosynthesis of amino acids, ABC transporters, mineral absorption, D-amino acid metabolism, and linoleic acid metabolism constituted the major metabolic processes across each group, as depicted in Figure 7F.

**Figure 8:**
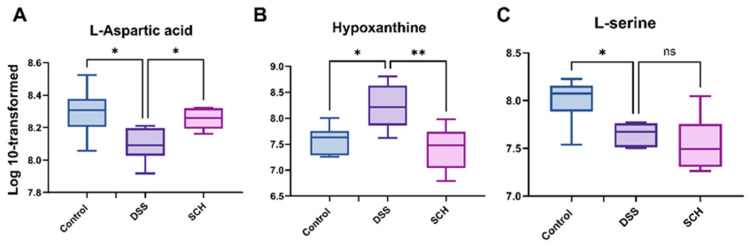
Effects of SCH administration on levels of (**A**) L-aspartic acid, (**B**) hypoxanthine, and (**C**) L-serine. * *p* < 0.05, ** *p* < 0.01, and “ns” denotes no statistically significant difference.

We combined the results from the random forest analysis with pathway enrichment findings and utilized box plots to illustrate notable intergroup variants. Our results indicated that aspartic acid, which was significantly reduced due to the administration of DSS, was significantly increased following SCH intervention (Figure 8A). DSS administration altered hypoxanthine levels in normal mice, however, SCH effectively reversed this trend (Figure 8B). Furthermore, regarding microbiological results, we speculated on the relationship between L-serine and *g-Akkermansia*, as well as *s-Escherichia coli*. According to the results presented in Figure 8C, DSS caused a significant decrease in L-serine levels in the intestinal tract of normal mice, which aligned with the microbiological findings of our study. Therefore, based on the metabolite results, it is suggested that SCH may promote the synthesis of anti-inflammatory substances, contributing to the alleviation of colitis.

### 2.7. Correlation Between Gut Flora, Metabolites, and Clinical Parameters

**Figure 9:**
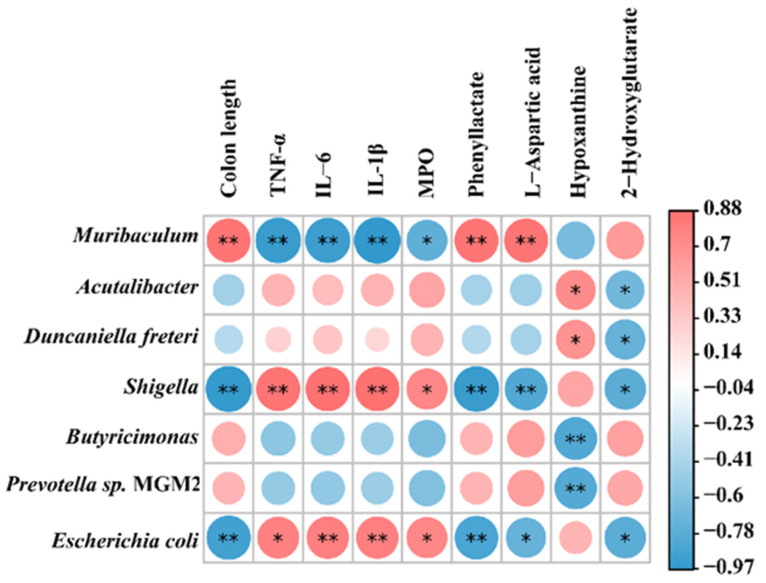
Correlation between gut flora, metabolites, and clinical parameters. * *p* < 0.05, ** *p* < 0.01.

We performed Spearman correlation analyses of colitis symptom parameters, microbiological results, and metabolite outcomes. The results were illustrated in Figure 9, which showed that the beneficial bacterium *Muribaculum* was positively correlated with colon length, as well as with the metabolites phenyllactate and L-aspartic acid. Conversely, it exhibited a negative correlation with MPO activity and pro-inflammatory factors, including IL-1β, IL-6, and TNF-α. In contrast, the harmful bacteria *Shigella* and *Escherichia coli* displayed results opposite to those of *Muribaculum*. In addition, when analyzing the correlation results concerning metabolites, hypoxanthine was positively correlated with *Acutalibacter* and *Duncaniella freteri*, while it was negatively correlated with *Butyricimonas* and *Prevotella* sp. MGM2. Additionally, 2-hydroxyglutarate demonstrated an antagonistic relationship with all the harmful bacteria identified in our results. These findings suggested that the harmful bacteria exhibited an antagonistic relationship, and as noted earlier, the alterations in gut flora and their metabolites may further elucidate the mechanisms by which SCH ameliorated DSS-induced colitis in mice.

## 3. Discussion

In the present study, a 3% DSS-induced colitis mouse model was utilized to investigate the effects of SCH at doses of 100 mg/kg, 200 mg/kg, and 600 mg/kg on body weight loss, DAI, colon length, splenic index, and the inhibition of pro-inflammatory factors in colitis mice, with all efforts demonstrating positive effects. To elucidate the mechanism of SCH protection, we combined macrogenomics with untargeted metabolomics for further exploration.

In terms of intestinal flora, the abnormal proliferation of *Acutalibacter* may contribute to an inflammatory response in the gut. It has been demonstrated that *Acutalibacter* activity and spontaneous inflammation in a mouse model resemble the manifestations of UC, suggesting a potential role for this bacterium in the pathogenesis of colitis [23]. Previous studies have indicated that the amount of *Oscillibacter* is reduced in mice treated with phloretin, and co-analysis has revealed that increased *Oscillibacter* levels correlate with elevated intestinal inflammatory markers, such as IL-6 and IL-1β [24]. Notably, the relationship between *Oscillibacter* and other intestinal bacteria is complex; *Oscillibacter* has been found to be positively correlated with *Colidextribacter*, consistent with the findings of the present study [25]. Furthermore, SCH treatment reversed this trend, highlighting SCH’s ability to ameliorate the shift in the gut flora of colitis mice towards that of control mice. Phylogenetic tree analysis indicated that the control and SCH groups exhibited a more similar species composition. Additionally, the SCH group demonstrated a high abundance of species that differed from the other two groups, including *Mucispirillum*, *Prevotella*, *Clostridium*, *Bacteroides*, and *Parabacteroides*. A healthy gut barrier is essential for preventing harmful substances and pathogens from entering the bloodstream, thereby mitigating the inflammatory response. Specific strains of *Parabacteroides* have been shown to enhance the integrity of intestinal epithelial cells and reduce inflammation associated with bacterial migration [26], suggesting the unique microbial composition of the SCH group and its potential role in healing inflammation.

A review discussing *Akkermansia muciniphila* as a next-generation probiotic noted that L-serine deficiency exacerbated DSS-induced colitis, leading to the proliferation of the pathogenic bacterium *Escherichia coli* in the inflamed bowel, which was accompanied by a sustained increase in the amount of *Akkermansia muciniphila*. This interaction synergistically promoted inflammation, providing a clear explanation for the high abundance of both *g*-*Akkermansia* and *s-Escherichia coli* observed in our DSS-induced mice. We can further elucidate these results by examining the amount of L-serine through metabolomics [27]. Notably, *s-Duncaniella freteri*, a newly identified species within the genus *Duncaniella*, is abundantly present in the intestinal tract of mice. A study investigating the efficacy and mechanism of action of live and heat-killed *Bacillus coagulans* BC198 as a potential probiotic for ameliorating DSS-induced colitis in mice also reported a high abundance of *s*-*Duncaniella freteri* within the DSS group [28]. Additionally, *g-Prevotella*, *o-Desulfovibrionales*, and *g-Lachnoclostridium* emerged as the major genera in the SCH group. *Lachnoclostridium* is known to produce short-chain fatty acids (SCFAs), especially butyrate, which is essential for maintaining gut health by serving not only as an important energy source for colon cells but also for promoting the growth and repair of colon epithelial cells [29]. Similar findings were reported in a previous study utilizing fecal microbiota transplantation (FMT) to alleviate experimental colitis in mice through the modulation of gut microbiota [30]. In conclusion, SCH can regulate and improve the composition of intestinal flora, increase the abundance of dominant flora, and promote the proliferation of beneficial bacteria, thereby alleviating inflammation.

Although *Shigella* is classified as a separate genus, genetic studies have demonstrated that *Shigella* species possess genomes remarkably similar to those of *Escherichia coli*. Some studies have suggested that *Shigella* species should be classified as distinct species within the genus *Escherichia coli*, indicating their close genetic relationship [31]. The SCH intervention reversed this trend, although the changes were not statistically significant. Furthermore, there was an observed increase in the amount of *Desulfovibrio* and *Acutalibacter*, alongside a decrease in the amount of *Butyricimonas* and *Prevotella* in the DSS group compared to the control group; however, these changes were also not significant. Notably, *Desulfovibrio* positivity has been significantly associated with the colon of patients suffering from acute and chronic UC [32]. Moreover, studies have indicated that *Desulfovibrio vulgaris* flagellin can promote the secretion of pro-inflammatory cytokines by interacting with LRRC19 proteins within host cells. This mechanism may lead to increased intestinal inflammation, thereby exacerbating UC [33]. The SCH intervention significantly reduced the relative amount of *Desulfovibrio*. Moreover, the SCH intervention significantly elevated the amount of *Butyricimonas*, which could produce butyric acid. Butyric acid is recognized for promoting intestinal epithelial cell integrity and enhancing the production of regulatory T-cells (Tregs), thereby suppressing excessive immune responses, which is critical in combating colitis [34]. While *Prevotella* is typically a harmless commensal, studies have indicated that *Prevotella copri* can exacerbate chemical-induced colitis [35]. Therefore, in this study, we aimed to confirm whether the harmful *Prevotella copri* was represented within the *Prevotella* genus. Our findings indicated that the SCH intervention significantly increased the relative amount of *Prevotella* and *Prevotella* sp. MGM2 at the species level compared to the DSS group. Regarding metabolic functions, *Prevotella* sp. MGM2 may play multiple roles in the gut, with studies suggesting its involvement in the production of short-chain fatty acids (SCFAs) that exhibit anti-inflammatory effects, especially butyrate [36,37]. In conclusion, the microbiological results demonstrated that SCH alleviated the disruption of gut flora and restored the gut bacterial composition in mice with UC.

In the results of gene function prediction, tuberculosis was identified as the metabolic pathway in the model group, while polyketide sugar unit biosynthesis emerged as the metabolic pathway in the SCH group. Previous research has demonstrated that children with mild or inactive disease exhibit significantly lower fecal lactoferrin concentrations compared to children with Crohn’s disease or moderate UC [38]. Interestingly, to better understand the gut microbiota of infants with cow’s milk allergy, it was found that lactoferrin was negatively correlated with polyketide sugar unit biosynthesis. This suggests that variations in lactoferrin levels may impact the synthesis of polyketide units, thereby affecting both the inflammatory state and microbial composition of the gut [39].

Results from metabolite analysis of gut microbes indicated that methylmalonic acid was an intermediate product in metabolic processes, primarily associated with vitamin B_12_ metabolism. A deficiency in vitamin B_12_ leads to an increase in the concentration of methylmalonic acid. Studies have demonstrated that patients with colitis exhibit a higher incidence of vitamin B_12_ deficiency, with a deficiency rate of 33% in patients with Crohn’s disease and 16% in those with UC. Several studies have investigated the relationship between methylmalonic acid and colitis. For instance, one study employed holotranscobalamin and methylmalonic acid to assess vitamin B_12_ status, revealing that intestinal inflammation independently affected cobalamin status. This finding suggested that methylmalonic acid served not only as a marker of vitamin B_12_ deficiency but also as a factor related to the pathological processes of colitis [40,41]. Following SCH intervention, three metabolites were significantly upregulated, including phenyllactate and 2-hydroxyglutarate, while twelve metabolites, including phenyl acetate, were significantly downregulated in DSS model mice. Phenyllactate may reduce inflammation by modulating the immune response. For instance, it has the potential to inhibit the production of pro-inflammatory cytokines, thereby mitigating intestinal inflammation. In models of IBD, phenyllactate has demonstrated promising anti-inflammatory effects, highlighting its significance in gut health. Additionally, certain intestinal bacteria, such as *Faecalibacterium prausnitzii*, have been observed to produce phenyllactate, with their metabolites closely associated with anti-inflammatory responses in the gut. These metabolites not only enhance the integrity of the intestinal barrier but also modulate the function of immune cells, further augmenting the anti-inflammatory effect [42,43]. Furthermore, 2-hydroxyglutarate inhibited the expression of pro-inflammatory cytokines induced by lipopolysaccharide (LPS), implying that in an inflammatory state, 2-hydroxyglutarate could attenuate the inflammatory response by reducing the levels of these molecules [44].

Amino acids play a crucial role in maintaining and enhancing intestinal barrier function, which is vital for preventing harmful substances and pathogens from entering the body. Research has demonstrated that specific amino acids can decrease the incidence of intestinal inflammation by strengthening the tight junctions of intestinal epithelial cells and reducing intestinal permeability. Notably, certain amino acids, such as aspartic acid, have demonstrated a positive effect in mitigating inflammation associated with amino acid-induced colitis [45]. Aspartic acid was found to ameliorate UC through the regulation of the RIPK pathway and modulation of gut flora composition in mice [46]. Patients with UC are at high risk for developing colon cancer (CRC), and it has been established that hypoxanthine plays a pivotal role in the metabolic network of these patients. Alterations in its metabolism may be associated with the progression of intestinal inflammation and the associated risk of cancer transformation [47]. In a study of delayed recovery from colitis due to pentachlorophenol (PCP) exposure, it was found that PCP exposure further exacerbated DSS-induced purine metabolism disorders, including an increase in hypoxanthine. In the present work [48], SCH intervention significantly decreased the relative intensity of hypoxanthine, suggesting a possible modulatory effect of SCH on DSS-induced purine metabolism disorders.

## 4. Materials and Methods

### 4.1. Reagents and Experimental Materials

Sea cucumber ovum was hydrolyzed with flavourzyme in an orbital shaking incubator (Senxin, Shanghai, China) for 5 h at an optimal temperature of 50 °C, a solid–liquid ratio of 1:17, and a pH of 7, with an enzyme dosage of 9500 U/g, while maintaining gentle agitation at 100 rpm. Upon completion of hydrolysis, the mixture was heated in a boiling water bath for 15 min to halt the reaction. After cooling the mixture in ice water, it was centrifuged at 10,000 rpm for 15 min at 4 °C, and the resulting supernatant was lyophilized to obtain the final SCH product. The DSS was acquired from Nanjing Dulai Biotechnology Co., Ltd. (Nanjing, China), while the fecal occult blood test kit was supplied by Shanghai Yuanye Biotechnology Co., Ltd. (Shanghai, China). Additionally, ELISA kits for mouse TNF-α, IL-6, IL-1β, and MPO were obtained from Nanjing Jiancheng Bioengineering Institute (Nanjing, China).

### 4.2. In Vitro SGD of SCH

The method referenced from Qiu et al. [49] was modified as follows: the composition of the simulated digestive fluid included simulated gastric fluid (SGF) (7.5 mL), which contained 2000 U/mL pepsin (1.6 mL), 0.3 mol/L CaCl_2_ (5 μL), and ultrapure water (695 μL), with the pH adjusted to 3.0 ± 0.1 using 1 mol/L HCl. For the simulated intestinal fluid (SIF) (11 mL), the components consisted of 800 U/mL trypsin (5 mL), 10 mmol/L bile salts (2.5 mL), 0.3 mol/L CaCl_2_ (40 uL), and ultrapure water (1.31 mL), with the pH modulated to 7.0 ± 0.1 using 1 mol/L NaOH.

### 4.3. Determination of Structural Characteristics and Physicochemical Properties of SCH and SCH-SGD

#### 4.3.1. Determination of Molecular Weight Distribution

Following the methodology outlined by Zhang et al. [50], the molecular weight distribution of SCH and SCH-SGD was determined using high-performance liquid chromatography (HPLC) (Agilent 1260, Santa Clara, CA, USA) equipped with a TSK Gel 3000 PWXL column (7.8 × 300 mm, Tosoh, Tokyo, Japan). The standards employed for constructing the relative molecular mass calibration curve included Gly-Gly-Gly (189 Da), GSH (307 Da), Gly-Gly-Tyr-Arg (451 Da), and VB_12_ (1355 Da).

#### 4.3.2. Surface Hydrophobicity

Surface hydrophobicity was assessed according to the method described by Kato & Nakai [51]. The samples were diluted with 0.1 mol/L phosphate buffer (pH 7.0) to a concentration of 1 mg/mL. Subsequently, 20 μL of 1-aniline-8-naphthalenesulfonate (ANS) (8.0 mmol/L, dissolved in 0.01 mol/L phosphate buffer, pH 7.0) was added to 2 mL of the samples and mixed thoroughly. The fluorescence intensity of each sample was measured at an excitation wavelength of 375 nm and a scanning wavelength of 400–650 nm using a fluorescence spectrophotometer (SHIMADZU, Tohoku, Japan).

#### 4.3.3. Solubility

The method for determining the solubility of proteins in hydrolysates was adapted from Liu et al. [52], with minor modifications. A solution with a hydrolysate concentration of 1% (*w*/*v*) was prepared, and the pH was adjusted to 2.0, 4.0, 6.0, 8.0, and 10.0 using 1 mol/L NaOH or 1 mol/L HCl. Following centrifugation at 4000 rpm for 20 min, the supernatant was collected, and the protein content was quantified using the method described by Lowry et al. [53]. The percentage solubility was calculated according to the following formula:

Solubility (%) = (Protein content of the supernatant)/(Protein content of the sample) × 100(1)

### 4.4. Animals and Experimental Design

Before being used for experiments, a group of 45 male C57BL/6j mice (6–8 weeks old) from Jinan Pengyue Laboratory Animal Breeding Co., Ltd. (Jinan, China) were acclimatized for 7 days to a 12-h light/dark cycle. The animal study protocol was approved by the Ethical Committee of Yantai University (protocol code 20231013s156, approved on 13 October 2023).

**Figure 10:**
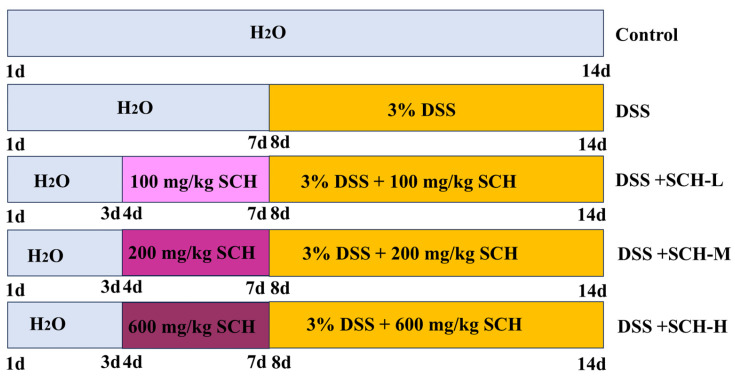
Schematic diagram of experimental design.

Subsequently, the 45 mice were divided into five groups, each consisting of nine mice: control, DSS, SCH low-dose (SCH-L), SCH medium-dose (SCH-M), and SCH high-dose (SCH-H). Oral doses of 100, 200, and 600 mg/kg were administered to the experimental groups over the course of the 14-day experiment (Figure 10). DSS was introduced into the drinking water to induce colitis and evaluate the impact of different SCH dosages on the condition.

The body weight of the mice was measured daily throughout the trial. On day 14, blood samples were collected from their eyes, and the mice were euthanized via cervical dislocation. Subsequently, the lengths of the colon and the weights of the spleen were recorded. The spleen index was calculated by dividing the spleen weight (in grams) by the body weight of the mouse (in grams). A section of the colon was immersed in a 4% paraformaldehyde solution for histological evaluation, while another section of the colon and its contents were extracted and stored at −80 °C.

### 4.5. Disease Activity Index (DAI)

After switching to a 3% DSS-inducing medication on day 8, fecal samples from the mice were collected daily at 2 p.m. and evaluated for fecal consistency and hemorrhage using a qualitative fecal occult blood test kit. The scoring criteria were illustrated in Table 2, where DAI was calculated as follows: DAI = (weight loss score + stool trait score + blood in stool score)/3 [11].

### 4.6. ELISA Assay

Serum was collected and analyzed for tumor necrosis factor-alpha (TNF-α), interleukin-6 (IL-6), and interleukin-1 beta (IL-1β) using ELISA kits. Additionally, 0.1 g of colon tissue was combined with 0.9 mL of saline in an EP tube. The mixture was homogenized at 15,000 rpm for one minute, divided into three batches with 30-s intervals, utilizing a tissue homogenizer (Potter-Elvehjem, VWRI432-0208, Malvern, PA, USA). At the conclusion of the homogenization process, the supernatant was collected and analyzed for myeloperoxidase (MPO) using ELISA kits.

### 4.7. Hematoxylin-Eosin (H&E) Staining of Intestinal Tissue

Colon tissue, fixed in paraformaldehyde, was excised, dehydrated, and sectioned to a thickness of 5 μm. The sections were mounted on clean glass slides, deparaffinized, rehydrated, and stained with hematoxylin and eosin, followed by assessment of colonic damage under a light microscope.

### 4.8. Gut Microbiota Analysis

Frozen mice colon contents stored at −80 °C were thawed, and macrogenomic DNA was extracted using the OMEGA Mag-Bind Soil DNA kit (OMEGA Bio-Tek, Narcross, GA, USA). The extracted DNA was quantified and assessed for quality using a Qubit™ 4 Fluorometer (Invitrogen, Waltham, MA, USA) and agarose gel electrophoresis. Metagenomic shotgun sequencing libraries, with an insert size of 400 bp, were constructed using the Illumina TruSeq Nano DNA LT Library Preparation Kit (Illumina Inc., Foster City, CA, USA). Sequencing was performed by Parsons Brinckerhoff (Shanghai, China), and the raw data were analyzed on the cloud platform at https://www.genescloud.cn/login (accessed on 15 October 2024).

### 4.9. Untargeted Fecal Metabolomics

Twenty-five milligrams of mice colon tissue were weighed in an EP tube at low temperature, combined with homogenizing beads, and added to 500 μL of an extraction solution containing an isotope-labelled internal standard (methanol: acetonitrile: water in a 2:2:1 volume ratio). The mixture was vortexed for 30 s, homogenized in a homogenizer at 35 Hz for 4 min, and then transferred to an ice-water bath for sonication for 5 min. This sonication process was repeated three times. The samples were then stored at −40 °C for 1 h. Following this, the samples were centrifuged at 4 °C at 12,000 rpm for 15 min, and the supernatant was collected in a feed bottle for online detection. An equal volume of supernatant from all samples was collected for quality control (QC) detection [54,55]. For the analysis of polar metabolites, a Vanquish ultra-performance liquid chromatograph (UPLC) from Thermo Fisher Scientific (Waltham, MA, USA) was employed to separate the target compounds using a Waters ACQUITY UPLC BEH Amide column (Waters, Milford, MA, USA) (2.1 mm × 50 mm, 1.7 μm). Liquid chromatography phase A consisted of an aqueous phase containing 25 mmol/L ammonium acetate and 25 mmol/L ammonia, while phase B was acetonitrile. The sample tray was maintained at 4 °C, and the injection volume was set at 2 μL. Subsequently, the data underwent pre-processing, experimental quality control, metabolite identification analysis, expression abundance analysis, and comparative analysis between two groups. Further details are available at https://www.genescloud.cn/login.

### 4.10. Statistical Analysis

Results were expressed as mean ± standard deviation (SD). Statistical analyses were performed using GraphPad Prism 9.00 software. Student’s *t*-test was utilized to assess differences between two groups, while one-way ANOVA was applied for comparisons among multiple groups. A significance level of *p* < 0.05 was considered statistically significant (* *p* < 0.05, ** *p* < 0.01, *** *p* < 0.001, **** *p* < 0.0001). All data included in this study were obtained from a minimum of three independent experiments.

## 5. Conclusions

Our study demonstrated that SCH was a polypeptide exhibiting notable stability following SGD, low surface hydrophobicity, and good solubility. Importantly, 98.21% of SCH molecules possessed a molecular weight of less than 1000 Da. Furthermore, SCH effectively ameliorated DSS-induced colitis in mice, as evidenced by a reduced DAI index and decreased intestinal damage in the UC mouse model. Furthermore, SCH lowered inflammation levels by decreasing the spleen index and the levels of pro-inflammatory factors. Utilizing macrogenomics and metabolomics techniques, this study elucidated the beneficial effects of SCH in ameliorating intestinal inflammation in colitis-affected mice. This was achieved by restoring the imbalance of gut microbiota, altering gut microbiota metabolites, and stimulating metabolic pathways, which included an increase in beneficial bacteria, such as *Muribaculum*, *Butyricimonas*, and *Prevotella* sp. MGM2, alongside a decrease in the relative amount of harmful bacteria, such as *Acutalibacter*, *Duncaniella freteri*, *Shigella*, and *Escherichia coli*. Metabolomics analyses further indicated that SCH exerted a more pronounced modulating effect on DSS-induced metabolic abnormalities, with the altered intestinal flora likely contributing to a reduction in the relative amount of hypoxanthine. This was coupled with an increase in the relative amount of metabolites, such as phenyllactate, 2-hydroxyglutarate, and L-aspartic acid, predominantly influencing amino acid metabolism to alleviate intestinal inflammation. However, due to the limitations of mouse models, clinical validation is crucial for advancing the practical application of SCH. In conclusion, SCH is positioned to potentially serve as an adjuvant therapy in the future treatment of colitis.

## Figures and Tables

**Table 1:** Molecular weight distribution of SCH and SCH-SGD.

Names	<500 Da (%)	500–1000 Da (%)	>1000 Da (%)
SCH	70.24	27.97	1.80
SCH-SGD	68.64	30.49	0.88

**Table 2:** Disease activity index (DAI) score.

Score	Weight Loss (%)	Stool Trait	Blood in Stool
0	<1	normal	normal
1	1–5	semi-sloppy stool	brown
2	5–10	loose stools	reddish
3	10–15	watery stool	visible traces
4	>15	watery diarrhea	gross bleeding

## Data Availability

The data presented in this study are available on request from the corresponding author.
